# Heterogeneity among melanoma databases and challenges in sustainability: A survey of the Melanoma Prevention Working Group

**DOI:** 10.1016/j.jdin.2024.02.001

**Published:** 2024-02-09

**Authors:** Sheyda Mesgarzadeh, Caitlyn N. Myrdal, Amanda H. Gong, Delaney B. Stratton, Brenna G. Kelly, Clara Curiel-Lewandrowski

**Affiliations:** a Division of Dermatology, Department of Medicine, University of Arizona College of Medicine-Tucson, Tucson, Arizona; b Department of Dermatology, University of Pennsylvania, Philadelphia, Pennsylvania; c Department of Dermatology, University of Utah, Salt Lake City, Utah

**Keywords:** challenges, database, development, heterogeneity, melanoma, skin cancer, sustainability

*To the Editor:* Melanoma is the fifth most common cancer in the US, with an estimated 100,000 new diagnoses of invasive disease and 8000 deaths in 2023.^1^ The remarkable, recent advancements in the management of melanoma emphasize the importance of melanoma databases to critically evaluate medical care, ensuring that disease staging, surveillance, and interventions reflect best practices.^2^ These databases are particularly relevant to address gaps in clinical management when clinical trials are not feasible.^3^ Given the variability in classification (and their overlapping features), we make a distinction between national cancer registries and nonnational databases (NNDs).^4^ National cancer registries are widely available for epidemiological and population research. In complement, NNDs typically collect more granular and customizable data not available in national cancer registries. Limited information exists regarding the development, content, and sustainability of melanoma NNDs.^5^ Therefore, we surveyed members of the Melanoma Prevention Working Group (MPWG), an international multidisciplinary collaboration of expert melanoma clinicians and researchers, to characterize the landscape and challenges in sustainability.

Following approvals by the institutional review board and the MPWG executive board, voluntary web-based surveys were distributed. Responses were collected from August 2021 to October 2021 with follow-up until September 2022. Descriptive analyses were conducted using Microsoft Excel (Microsoft Excel) and PRISM-GraphPad (GraphPad Prism).

Among the 83 institutions represented within the MPWG, 28 (response rate of 34%) completed the survey. Of these, 16 (57%) maintained an active database, 4 (14%) an inactive database, and 8 (29%) did not have a melanoma database at their institution (Table I). Most databases capture demographic data (95%), histological information corresponding to initial biopsy (90%), surgical excision (85%), and staging (90%); whereas, genetic testing (50%), participation in clinical trials (50%), and surveillance (45%) are less frequently documented. The most significant barriers to database development, rated on a 10-point Likert scale from 1 (no challenge) and 10 (most challenging), were resources (6.8) and time (6.7). Among those with an active database, inputting new (56%) and updating records (56%), and the number of staff involved (56%), were the most reported challenges of database maintenance. In comparison, inputting new (75%) and updating old records (75%), number of staff involved (75%), funding (75%), time (75%), and institutional support (75%) represented the most common reason for database termination.

**Table I:** Database characteristics and challenges associated with database maintenance

Database status (*N* = 28)	No (%)
Active	16 (57)
Inactive	4 (14)
No database	8 (29)
Platform (*N* = 20)	No (%)
REDCap	7 (35)
Excel	5 (25)
SQL	5 (25)
Unknown	3 (15)
Demographics by country (*N* = 20)	No (%)
United States	18 (90)
Australia	2 (10)
Scope of database (*N* = 20)	No (%)
Melanoma in situ (Tis) and/or invasive melanoma (T1a or greater)	13 (65)
Invasive melanoma (T1a or greater) only	3 (15)
Melanoma in situ (Tis) only	0 (0)
Metastatic melanoma (*N*1a or greater and/or M1a or greater) only	1 (5)
Other∗	2 (10)
Unknown	1 (5)
Active/inactive database recorded factors (*N* = 20)	No (%)
Demographics	19 (95)
Initial pathology (biopsy)	18 (90)
Staging	18 (90)
Surgical pathology	18 (90)
Surgery	17 (85)
Recurrence	15 (75)
Metastasis	14 (70)
Risk factors	13 (65)
Imaging	13 (65)
Neoadjuvant therapy	13 (65)
Adjuvant therapy	13 (65)
Active therapy	13 (65)
Response to therapy	11 (55)
Clinical trials	10 (50)
Genetic testing	9 (45)
Surveillance	9 (45)
Other†	2 (10)
Database size by number of records (*N* = 20)	No (%)
0-99	0 (0)
100-499	2 (10)
500-2499	5 (25)
2500-12,499	5 (25)
12,500-62,499	4 (20)
Unknown	4 (20)
Barriers to development (1 = no challenge, 10 = most challenging)	Median/mean
Financial (*N* = 9)‡	5.7/5
Number of personnel (*N* = 10)	6.2/5
Time (*N* = 13)‡	6.7/7
Experience (*N* = 9)	6.2/6
Resources (*N* = 11)	6.8/7
Other§	5/5
Barriers to maintenance (*N* = 16)‖	No (%)
Inputting new records	9 (56)
Updating old records	9 (56)
Number of staff involved	9 (56)
Funding	8 (50)
Time	6 (37)
Ease of use	2 (12)
Reliance of data extraction	7 (44)
Institutional support	5 (31)
Other¶	1 (6)
Reasons for database termination (*N* = 4)#	No (%)
Inputting new records	3 (75)
Updating old records	3 (75)
Number of staff involved	3 (75)
Funding	3 (75)
Time	3 (75)
Ease of use	1 (25)
Reliance of data extraction	2 (50)
Institutional support	3 (75)
Other∗∗	1 (25)

*REDCap*, Research electronic data capture; *SQL*, structured query language.

∗ Patients with all levels of melanoma risk, patients without history of melanoma, and familial melanoma.

† Radiation therapy, biopsy type, and location in state.

‡ Single participant excluded for not rating barrier to development on Likert scale.

§ Evolving technology reported as barrier to development by single participant.

‖ Active databases only.

¶ Changing priorities.

# Inactive databases only.

∗∗ Participant reported leaving institution as reason for database termination.

Fig 1 demonstrates the considerable variability in maintenance with a greater proportion of more frequent entry of new records (filled bars) and updating of existing records (unfilled bars) among progressively larger NNDs.

**Fig 1:**
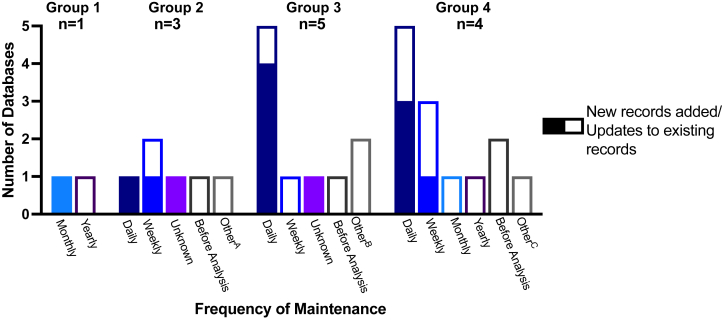
Frequency of database maintenance relative to total number of database records. Database size was divided into 4 groups by total number of records. Group 1: 100 to 499 records (*n* = 1); group 2: 500 to 2499 records (*n* = 3); group 3: 2,500 to 12,499 records (*n* = 5); group 4: 12,500 to 62,499 records (*n* = 4). **A,** Records updated at follow-up visits at 6, 12, or 24 months. **B,** Semiannual updates or not updating due to lack of funding. **C,** Quarterly updates.

These findings offer insights into the current landscape of NNDs, revealing the variability in database content, likely reflecting individual priorities among institutions, and operational challenges in their development and sustainability. Study limitations include restriction to MPWG-affiliated institutions and response bias. Further studies that reflect a broader cohort of databases, particularly at the international level, are necessary to capture a comprehensive representation.

An improved understanding of available NNDs unveils new avenues for interinstitutional collaboration and data sharing to encourage more standardized data collection. This study serves as a reference to actualize these opportunities and mitigate future obstacles to support robust, sustainable databases to address critical gaps in the melanoma field.

## Conflicts of interest

None disclosed.
